# Polypyrrole Effect on Carbon Vulcan Supporting Nickel-Based Materials Catalyst During Methanol Electro-Oxidation

**DOI:** 10.3390/ma19030523

**Published:** 2026-01-28

**Authors:** Alfredo Salvador Consuelo-García, Juan Ramón Avendaño-Gómez, Arturo Manzo-Robledo

**Affiliations:** Sección de Estudios de Posgrado e Investigación, Departamento de Ingeniería Química, Escuela Superior de Ingeniería Química e Industrias Extractivas, Instituto Politécnico Nacional, UPALM, Zacatenco, Av. Instituto Politécnico Nacional S/N, Ciudad de México 07738, Mexico; alfredosalvadorclgarcia@hotmail.com

**Keywords:** polypyrrole (PPy), methanol oxidation reaction (MOR), direct methanol fuel cell (DMFC), carbon-based catalyst, nickel hydroxide, electric conduction enhancement, catalytic activity, redox interactions, low charge transfer resistance, high turnover frequency (TOF)

## Abstract

The catalyst in methanol oxidation plays a pivotal role in direct fuel cell reaction. The aim of this work is to study the influence of polypyrrole polymer (PPy) added in the carbon Vulcan support for the methanol oxidation reaction. The catalytic active phase synthesized was nickel-based materials, which have been demonstrated to exhibit remarkable chemical stability in alkaline solutions. The metallic-active phase was supported at the PPy-carbon Vulcan matrix. PPy is a conductor polymer and the research of electric conduction in synergy with a carbon Vulcan and a Ni catalyst is scarcely reported. The morphology characterization of composite catalytic material was carried out by XRD, XPS, and TEM techniques. In turn, the catalytic activity of the composite is characterized by means of cyclic voltammetry (CV). Electrochemical impedance spectroscopy (EIS) showed the influence of PPy on the charge transfer resistance (Rch. t.). The results indicate that a decrease in the Rch. t. was associated with an increase in methanol oxidation; therefore, higher amounts of charge transfer is produced. Furthermore, the DEMS technique corroborates the EIS results, confirming elevated conversion toward oxidation products. In turn, the selectivity of the composite-catalytic support on the methanol oxidation was elucidated using in situ Raman spectroscopy.

## 1. Introduction

The depletion of fossil fuels has prompted the exploration and development of alternative energy sources. Fuel cells represent a promising solution, as they facilitate the efficient conversion of chemical potential energy into electric energy. Notably, this process occurs without the emission of greenhouse gases [1,2]. Direct methanol fuel cells remain a subject of extensive research due to their advantageous properties. Methanol is a renewable fuel with safer operation compared with hydrogen fuel cells. It is noteworthy that the high volumetric energy density of this fuel can be transformed either by oxidation in an alkaline or an acidic environment. Moreover, the availability of methanol from renewable resources such as crop reserves or biomass waste facilitates the fulfillment of criteria associated with a circular economy and environmentally friendly processes [3]. The design of direct fuel cells operating with liquid methanol requires only room temperature and ambient pressure. All these advantages make direct fuel cells a potentially useful technology with applicability in portable electronics [4].

The use of alkaline electrolytes increases durability of fuel cell components, where OH- ions are transferred from cathode to anode. Moreover, it is easy to adapt alkaline fuel concentrations to minimize system degradation, and to inhibit carbon support dissolution, or to avoid anodic catalyst leaching. The use of catalysts free of noble metals is an additional advantage because it confers moderate alkalinity. Evidence suggests that the effect of nickel or carbon-supported nickel composites is gaining relevance in the methanol electro-oxidation reaction [4,5,6].

For example, catalytic activity at Ni/C electrodes in methanol oxidation reaction is subject to variation according to the amount of nickel deposited at an applied potential. A previous activation of the catalyst shows that methanol oxidation starts when nickel oxide is formed on the electrode surface. Reaction occurs toward direct oxidation with NiO(OH) for thin oxides and charge transference for thick oxides. The NiO(OH) accumulation provokes an inhibitory effect on the catalyst activity; however, such an effect can be counteracted with a reactivation period on the catalyst [6].

Polypyrrole (PPy) has been studied since 1963 when McNeill et al. reported initial knowledge of conductive properties [7]. Particularly, PPy is an electric conducting polymer with good mechanical, chemical, and thermal resistance depending on its molecular weight [8]. The aromatic character of the unsaturated ring and the basic nitrogen sites induce excellent electron and proton conductivity. Therefore, the addition of PPy polymer added to the catalyst support carbon Vulcan could enhance mass and charge transfer interactions.

On the other hand, a novel procedure for the synthesis of PPy was proposed to synthesize PPy following a green method, avoiding the use of FeCl_3_. The resulting PPy-based polymer exhibited a high molecular weight with good stability in different pH-environments [9]. On the contrary, a more reactive PPy can be obtained by electrochemical methods, usually characterized by short polymer chains averaging 8-unit pyrrole rings. However, the low molecular weight confers on it also a lower stability, which can lead to depolymerization under even mild chemical and thermal conditions [9].

As stated previously, electron and proton conductivity is a property of practical interest in catalyst supports, including polymer-based materials, such as PPy. A review of the literature suggests that increasing the size of the polymer chain, and consequently the order of electronic conductivity, is a matter of its stability [10]. In this context, the novel synthesis method herein developed revealed good polymer stability and synergistic electro-chemical behavior of PPy and carbon Vulcan.

The aim of this work is to present novel electro-catalytic findings concerning the interaction between PPy and the utilized carbon Vulcan which functions as a support for the Ni-based catalyst. The initial step in anchoring the polymer onto the carbon black surface involves impregnating the polymer chains with Al(OH)_3_. For this case, aluminum acts as a Lewis acid on the nitrogen atoms available along the polymer chain, facilitating the anchoring process [11]. The catalytic composite material modified with nickel species was evaluated toward the methanol oxidation reaction for direct fuel cell applications. Furthermore, the obtained materials were characterized using techniques such as XRD, XPS, SEM, TEM, CV, EIS, CA, and DEMS-RAMAN spectroscopy in situ.

## 2. Materials and Methods

### 2.1. Chemicals

Carbon Vulcan XC-72R from Fuel Cell Store (Sigma-Aldrich Corporation, St. Louis, MO, USA) was employed as catalyst support. Pyrrole 98.0% purity reagent grade from Sigma-Aldrich (Sigma-Aldrich Corporation, St. Louis, MO, USA) was used as received, whereas iodine and NiCl_2_ at 98.0% purity reagent grade were obtained from Sigma-Aldrich. HCl and NaOH were purchased from Meyer (Myers Industries, Inc., Akron, OH, USA). The AlCl_3_ was prepared by means of the chemical reaction between HCl 1.0 M and the corresponding stoichiometric weight of aluminum foil. The resultant solution of AlCl_3_ was prepared for a 1.0 M concentration of aluminum salt. All solutions were prepared using deionized water at 18 mΩ resistivity. The surface of the catalyst support was chemically functionalized in independent compositions. The first composition contains only the metallic-catalyst active phase. The second catalyst support is covered by Al(OH)_3_ with PPy anchored to it. In this formulation, one aluminum atom is linked to one nitrogen atom from PPy.

### 2.2. PPy Synthesis

The PPy polymer is synthesized using pyrrole as a precursor monomer. In the first step, a thin film of deionized water is placed on the bottom of a beaker, then the pyrrole is expanded on the surface of the water layer. Thereafter, 1.0 mL of pyrrole was reacted with 4.0 g of solid I2, without external stirring. The resulting mixture underwent a rapid transformation, giving a dark-colored solid. The structural configuration of the obtained solid is consistent with that of iodine-pyrrole dimer (IPD).

The iodine-pyrrole dimer is then homogeneously distributed on the bottom of an Erlenmeyer flask connected to a second similar Erlenmeyer flask by means of a pyrex glass pipe. The Erlenmeyer flask containing the iodine-pyrrole is put on a heating grid, the other one stays apart. Pyrolysis of iodine pyrrole dimer is produced by heating at 400 °C; during thermal oxidation of iodine pyrrole dimer, a violet gas of iodine is even visible, and it flows from the reaction Erlenmeyer to the receiving Erlenmeyer. The reaction stops in 15 min. To enhance the iodine detachment in the remaining dark solid, the dark polypyrrole polymer is let in the reaction flask for 1.5 h. The dark solid is the polypyrrole (PPy), that is a black hard solid and similar in appearance to graphite at high molecular weights [7,8]. When pyrolysis is finished, the PPy is grinded on a porcelain mortar until the thinnest dust is observed. Eventually, this dust is stored for further analysis and synthesis of materials.

### 2.3. Catalyst Synthesis

The catalytic supports were prepared and analyzed in two types. The first catalytic support only contains the catalytic active phase being the Ni(OH)_2_; it is supported on carbon Vulcan, and the content of Ni(OH)_2_ was in turn studied at two different mass percentage amounts of 8.0 wt. % and 10.0 wt. %. The second catalytic support material was synthesized, anchoring the polypyrrole polymer PPy to the carbon Vulcan. The role of the Al(OH)_3_ in this formulation is to function as an intermediary molecule to allow the anchorage of the PPy polymer on the carbon Vulcan support. In both types of supports, a relation of 90% of carbon black is impregnated with 10 wt. % of Ni(OH)_2_, for the type 1, and 10 wt. % of Al(OH)_3_, Ni(OH)_2_, and PPy as a whole set.

For the first support, a hybrid between impregnation and coprecipitation methods was followed. Initially, a certain amount of carbon Vulcan was put into a rounded bottom flask of a rotary evaporator. After that, a solution of known concentration of NiCl_2_ was introduced into the flask. The impregnation of the active phase is carried out in a rotary evaporator during 90 min at 80 °C, the ballon containing the mixture of carbon Vulcan and metallic hydroxides is programed to turn at 200 rpm and changing the rotation intermittently clockwise and opposite direction each 30 s for 90 min. This synthesis system was heated at 80 °C, using an automatic water-temperature-controlled bath. Eventually, the mixture was filtered using a suction assisted system to obtain a wet carbon material that was washed with a 1.0 M NaOH solution, and then with enough deionized water to eliminate the resulting NaCl and NaOH excess. After hydroxilation, only α-Ni(OH)_2_ was expected [12], confirmed later through XRD and XPS tests. The wet solid was dried with room temperature and pressure air (24 °C, 585 mm Hg, average) for 24 h, and the final dry solid was stored for further analysis.

For materials containing only Ni(OH)_2_, the concentration of NiCl_2_ precursor solution was calculated using the following formula,

(1) $$\%f=C_{nc}\cdot {MW}_{nh}\cdot R\times 100$$

where %f is the set up active phase, C_nc_ is NiCl_2_ concentration, MW_nh_ is Ni(OH)_2_ molecular weight, and R is the volumetric amount of water that can adsorb a certain mass of employed carbon Vulcan. For this case, R = 2.0 μL/mg. This equation follows a mass balance considering R as a constant for any of the precursor solutions. In fact, only a certain quantity of humidity is required to produce impregnation on a carbon sample and further coprecipitation that converts impregnated NiCl_2_ into Ni(OH)_2_.

For materials containing PPy, the material balance was applied with the particularity that PPy and Al(OH)_3_ are in a tri-molar weight relation, it means that for every atom of Al, 3 atoms of N proceeding from PPy were added. This must be matched with the fact that both NiCl_2_ and AlCl_3_ precursors were in the same impregnation solution, simultaneously adsorbed by the carbon Vulcan, in every case considering R as a constant. The amount of PPy calculated for addition was made before carbon Vulcan was put inside the rotary vapor flask, just to avoid uncertainty while weighing these solids. Afterwards, the synthesis procedure was the same as the employed in the support material containing only Ni(OH)_2_.

As PPy is a non-reactive and insoluble substance at a very wide range of temperatures and pressures, it also presents no adhesion to carbon Vulcan. According to cyclic voltammetry tests, this was associated with high charge transfer resistances and low adsorption activity. The PPy is a basic Lewis-site rich polymer, the use of an Al compound, such as Al_2_O_3_ or the selected Al(OH)_3_, was chosen to serve as an intermediary molecule on the carbon Vulcan surface to the anchorage of PPy.

### 2.4. Characterization

The way methanol consumption evolved was observed using Differential Electrochemical Mass Spectroscopy (DEMS), observation was made through in situ Raman spectroscopy in real-time ongoing reaction. Raman spectroscopy analysis was carried out using a BWSpec 4.10 spectrometer (Thermo Fisher Scientific Inc., Waltham, MA, USA) at 785 nm laser wavelength, varying its power level and the integration times to reach appropriate spectra intensities. Whereas XRD patters were obtained with a step of 5 °/min, from 5° to 100 °, and with a 300 W/600 W 40 kV energy Kα wave Sealed Tube-B Cu lamp D/tex Ultra Detector monochromator (Rigaku Corporation Tokyo, Japan), at a 1.54 Å wavelength. On the other hand, a XPS Thermo Fisher Scientific photoelectron spectrometer (Thermo Fisher Scientific, Waltham, MA, USA) with a K-α monochromatized was employed, using a 400 μm spot size at 200 and 1486.6 eV Al Kα X-ray source with a 20 eV pass energy, obtaining a high-resolution spectra. For TEM analysis a TITAN 80e300 (Thermofisher, Waltham, MA, USA) microscope at 300 kV was used. To prepare the samples, slow evaporation of a drop of purified colloidal solution onto a carbon-coated copper grid took place.

The electrochemical analysis was carried out by means of cyclic voltammetry (CV); a droplet of ink is deposited on the front face of the working electrode (WE). The ink composition was a dispersion of 1 mg/214 μL containing isopropanol, water, Nafion polymer and the material of interest to be analyzed. The dispersion was produced using ultrasonic waves in a water bath. The sample was scanned by means of an electric potential window ranging from −0.5 V to 0.525 V at a 0.050 V/s scan rate, during 30 cycles. The very first scan is carried out for activation of the sample. Then, the electrode with the catalyst support was put again in the presence of KOH and methanol to develop methanol oxidation by electrochemical reaction. The experimental conditions for methanol oxidation were run at the same potential window and scan rate, during 5 cycles. A process alike was employed to calculate ECSA: the same kind of WE initially activated in a 1.0 M KOH solution was introduced in a 10 mM 4[Fe(CN)_6_] and 1.0 M KCl solution. From −0.20 to 0.65 V CV was developed during 5 cycles, using scan rates from 5 to 200 mV/s. Appropriate calculations following the Randles-Sevcik electrochemical diffusion theory permits us to know the aimed property.

Electrochemical impedance spectroscopy (EIS) was carried out strictly and systematically with a similar approach starting without methanol and following the described activation steps. The EIS begins without methanol, in a further stage of the assessment methanol is incorporated. The KOH and methanol concentrations are the same as those stated in the CV assessment: the reference potential of 0.375 V—the starting potential of the charge transfer process—, frequency variations starting from 105 Hz and ending to 0.1 Hz, and an RMS potential of 10 mV. It is noteworthy to mention that the CA technique as well is carried out with the same activation steps; the electrochemical cell was submitted to a constant 0.375 V potential during 7200 s, after 15 s previous steps at 0.1, 0.2, and 0.3 V to reach the final potential without generating overloads.

Several results can be obtained according to the measurements registered with the operating potentiostat. One of the aspects obtained from CV data was the peak current. Another CV extracted information was the catalytic activities since the CV profile was integrated and related to the reacting mass. On the other hand, EIS data were interpreted by comparison against an impedance model where its corresponding electric circuit is arranged to fix as much as possible the experimental data with the impedance profile. Such a circuit gives information about the electrochemical system conceived within the testing cell, including solution resistance, capacitive reactance, and charge mass transfer interactions. The calculations for numerical fitting were made using a brute force method, adapting its capabilities to some automatization structures that permitted to deal with the data in batch mode [13].

## 3. Results and Discussion

### 3.1. X-Ray Diffraction

**Figure 1:**
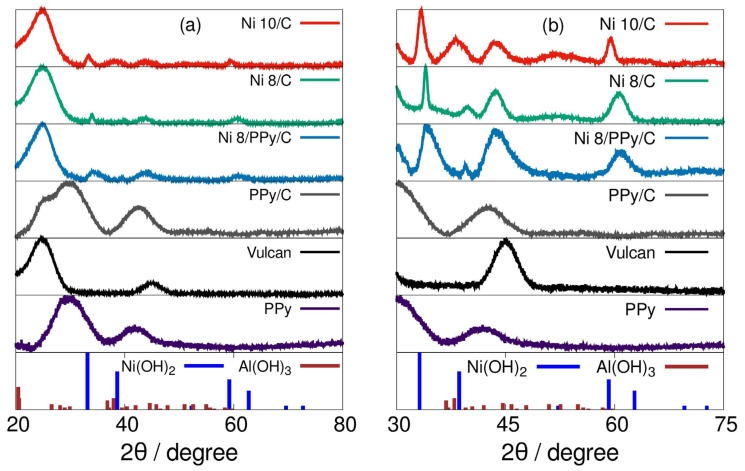
Diffractograms correspond to catalysts containing PPy, at different Ni(OH)_2_ active phase compositions—excepting Ni 10/C (10 wt. %)—and to free synthesized PPy through IPD pyrolysis. In dark blue the Ni(OH)_2_ chart and in brown the corresponding to Al(OH)_3_ [14]. In (**a**), from 20 to 100°; in (**b**), a close-up from 30 to 75°. From here and after, materials are Ni X/S, with X indicating the mass percentage of present Ni(OH)_2_ and S the supporting solids.

Catalysts diffractograms are displayed in Figure 1. The peaks corresponding to Ni(OH)_2_ are located at ca. 33° and are shifted to right as PPy amount increases [14]. The amorphous nature of PPy and the carbon Vulcan particles are both identified in diffractograms [15]. The lack of crystallinity in PPy is not a limiting characteristic for the electric conductivity as is shown by EIS results. In addition, TEM images showed that polymer chains conforming to the PPy macromolecule are aligned linearly in some domains inside the bulk particle. The longer the chain, the easier the electron flow through polymeric chains [16]. From XRD the predominant phase related to nickel is Ni(OH)_2_ as active site for redox process, see below and Section 3.3.

According to diffractograms, τ crystallite sizes were calculated using Scherrer model, Equation (2) where λ is the X-ray wavelength, β is the line broadening at half the maximum intensity, θ is the Bragg angle and K is a dimensionless shape factor equals to 0.9 [17].

(2) $$\tau =\frac{K\cdot \lambda }{\beta \cdot cos(\theta )}$$

Table 1 shows the crystallite sizes given by 8.0% and the 10.0% Ni(OH)_2_ catalysts. In this sense, chemical stability of the referred materials is linked to their crystallite sizes.

The crystallite even at small size can also be structured in layers beneath the main active phase particle. Each layer can be degraded at a time; then, the more layers exist, the less corrosion-susceptible the particle is.

### 3.2. SEM Analysis

**Figure 2:**
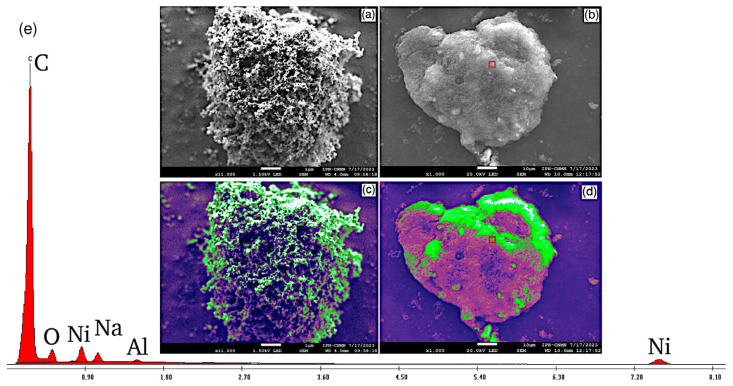
Ni 8/PPy/C micrographs containing PPy distinguishing (**a**) catalyst porous surface, and (**b**) supported phase on carbon Vulcan, the red square presents the analysed surface of the sample in the SEM assesment. The (**c**,**d**) images indicate differentiation of compounds conforming the catalyst: white colored corresponding to nickel hydroxide; green regions associated with aluminum hydroxide; purple-magenta regions indicate carbon black presence and dark regions are set for pores; and (**e**) shows the elemental analysis found in the sample.

In Figure 2, scanning electron microscopy (SEM) micrographs and energy dispersive X-ray spectroscopy (EDS) analysis corresponding to Ni 8/PPy/C sample can be observed. The Figure 2a image allows verification of high porosity, due to the presence of carbon Vulcan. Porosity allows to develop high surface areas which in turn contribute to higher catalytic activity, most of the surface is likely to be occupied by catalytic active layers. The Figure 2b image contains another catalyst particle of greater size than the former. In this case the region in the red square was selected to make EDS analysis. The result of this is in Figure 2e, where all chemical species in the catalyst support were found, excepting nitrogen N. It is important to highlight the fact that the PPy is present in a very low mass percentage. Therefore, the probability of finding a particle of PPy is the lowest compared to seeing a particle of carbon Vulcan. The Figure 2c,d images are color differentiated to facilitate the identification of chemical species in the support catalyst.

In both colored images impregnated materials can be appreciated over the carbon Vulcan surface. Micrograph in Figure 2c shows a well-distributed impregnation than in Figure 2d. This can be attributed to their differences in surface geometry and sizes. For the smaller carbon particle, porosity is more present and consequently the dispersion of catalytic active phase is more effective. On the other hand, the porosity in the bigger carbon particle is not so evident; nevertheless, this may be due to differences in scale since figure Figure 2a is 10 orders smaller than figures Figure 2b,d.

### 3.3. XPS Analysis

**Figure 3:**
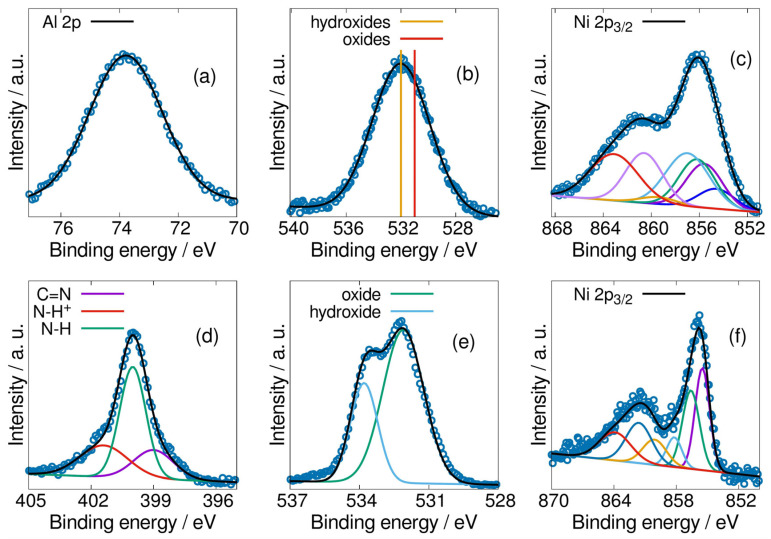
XPS spectra obtained for Ni 8/PPy/C catalyst. The binding energies are in (**a**) for Al, (**b**) for O, and (**c**) for Ni. In (**d**), the characteristic N binding energies for PPy. Ni 10/C is shown in (**e**) for oxygen and (**f**) for nickel.

Catalyst Ni 8/PPy/C was analyzed using XPS. As expected, in Figure 3a can be seen Al^+3^ oxidation state from its XPS spectrum corresponding region at 74.0 eV binding energy, because it is the only possible apart from its elemental state [18]. Nevertheless, differences can be stated between its hydroxide and oxide forms. Thus, oxygen region in XPS between 530 and 534 eV may include either hydroxide or oxide, or both binding energies. For hydroxides, oxygen binding energy is 532 eV; for oxides, binding energy is 531 eV [18]. XPS spectrum for catalyst at (b) shows a 532 eV binding energy, confirming the material is constituted only by hydroxides, including those of aluminum and Ni(OH)_2_. Additionally, Ni^+2^ oxidation state is confirmed in spectrum (c) and no Ni^+3^ was detected [19].

The XPS results enable us to state that predominant nickel species in the catalyst are assigned to nickel hydroxides; in turn such a result is in accordance with the synthesis method employed during the co-precipitation step [12]. The coprecipitation method was chosen because it produces Ni(OH)_2_ from the precursor NiCl_2_. Indeed, co-precipitation is an integral component of the catalyst design process. The aim of the co-precipitation was firstly to synthesize catalytic support able to resist corrosion, and secondly, such a support be reactive in electrochemical oxidation of methanol. In this context, the presence of OH- ions within metallic atoms may also contribute to corrosion resistance protection. Moreover, some mechanical and thermal stability, as indirectly was verified during cyclic voltammetry experiments, may be attributed to the presence of hydrogen bonds within the crystalline structure of the hydroxide species [20]. A similar result was observed for catalyst Ni 10/C in Figure 3f. However, in the oxygen high-resolution spectrum, oxidized species are dominant with respect to hydroxides species, see Figure 3e.

The competition between oxidized and hydroxides species confers on the 10% metal-active phase a chemical behavior of lower stability at the alkaline environment. In summary, the outlined hydroxylation method has been demonstrated since the synthesis was designed to obtain the redox oxidation states.

Following this analysis, the spectrum corresponding to PPy is displayed in Figure 3d with its corresponding curve fitting. The determined C=N, NH+, and NH groups are critical to the definition of PPy. Specifically, the NH group is the predominant nitrogen structure present in pyrrole rings, while C=N and NH+ are observed due to its resonance. It is important to note that, given the presence of the polymeric form but not the individual ring form, the characteristic resonance structures refer to the electric conduction process of deallocated electrons occurring in the macromolecular chain [9]. This phenomenon has been shown to improve charge transfer within catalyst Ni 8/PPy/C.

### 3.4. Transmission Electron Microscopy TEM

**Figure 4:**
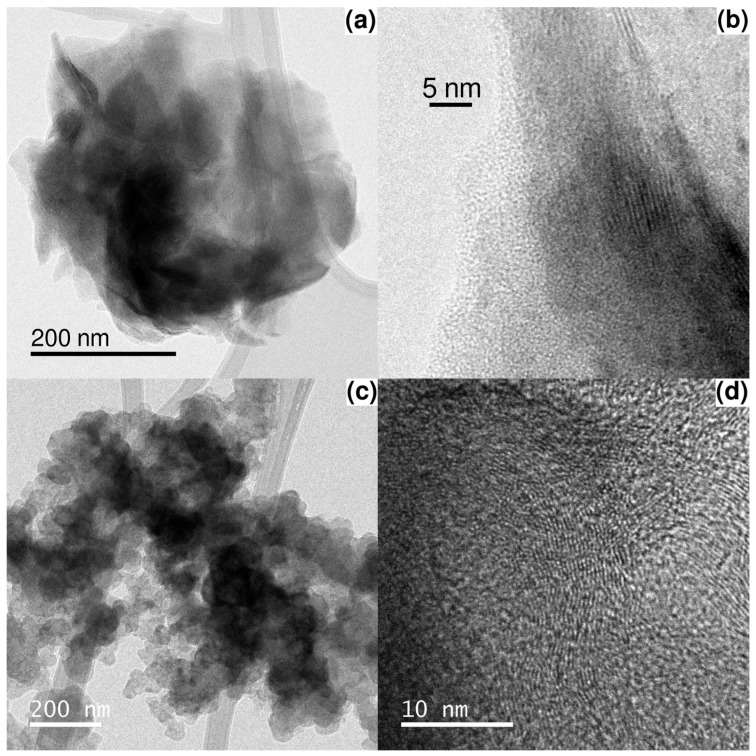
TEM PPy particle images, (**a**) 200 nm scale and (**b**) 5 nm scale. Left micrography shows a complete view of the particle, whereas a zoom is shown in the right micrography. The right micrography reveals a pattern of parallel linear chains which are the polymeric molecules of PPy. Images of catalyst Ni 8/PPy/C are shown in micrography (**c**) 200 nm scale. The micrography (**d**) 10 nm scale allows to distinguish linear chain of PPy with random trajectories of growth.

As illustrated in Figure 4a,b, PPy-intrinsic properties were also studied using TEM. In this case, as electric conductivity depends on its macromolecular chain length, which is determined by its polymerization grade, it is pertinent to observe that the polymer molecules were distinguished by means of a pattern of parallel lines. This observation was made during the TEM procedure which yielded 200 nm scale images. In addition, those patterns were identified within the 5 nm scale.

As demonstrated elsewhere, the length of polymeric chains is directly correlated with their electric conductivity [9]. It is noteworthy to mention that polymer chain macromolecules exhibit linear continuity, such a characteristic foster electron movement under electric field changes. Furthermore, the enhancement of charge transfer due to ordered polymer chains contributes synergistically to improving the methanol oxidation carried out by the Ni phase.

For catalyst Ni 8/PPy/C, some differences between PPy and carbon Vulcan can be highlighted, as shown in Figure 4c,d: First, the molecular arrangement patterns directly affect their charge-transfer mechanism as demonstrated from EIS experiments. Second, it has been established that electrons and electric fields do not exhibit a restriction in terms of the number of physical pathways, enhancing their availability for redox process.

### 3.5. Cyclic Voltammetry CV

**Figure 5:**
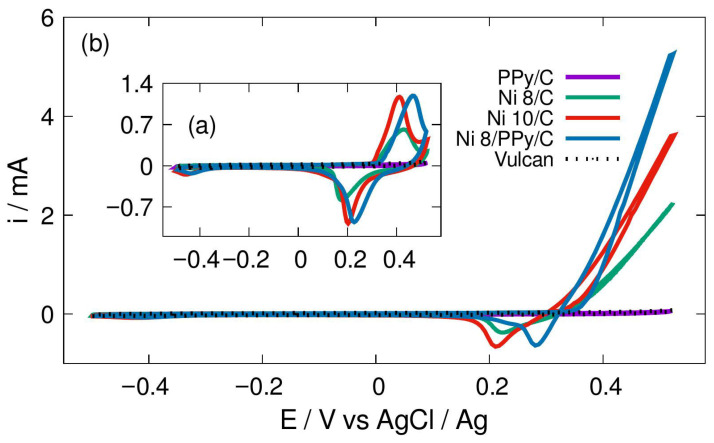
Active-phase-materials CV profiles (**a**) without methanol, in 1.0 M KOH and (**b**) in 1.0 M KOH and 0.5 M methanol. Scan rate of 50 mV/s.

The unmodified support-carbon matrix (Ni 8/C, Ni 10/C, C) and modified carbon support with PPy (Ni 8/PPy/C, PPy/C) were put on cyclic voltammetry assessments. Such technique allows us to measure the current versus potential behavior of the studied materials. The CV tests in alkaline environment (1.0 M KOH) with no methanol, then in presence of methanol (at 0.5 M) are shown in Figure 5. In a first approach, notice that redox processes associated with nickel species were observed in solution without methanol, Figure 5a, as expected [4].

The redox process increases with respect to the amount of nickel on the matrix free of PPy, Figure 5a. Interestingly, the faradic current associated with methanol oxidation also increases as a function of the nickel content, Figure 5b, for both PPy-modified and non-modified materials. However, notice that such current is higher when compared to the samples free of PPy, Figure 5b. The catalytic effect is also validated since (i) at carbon-PPy samples free of nickel-phase, no redox processes associated with methanol oxidation were observed, and (ii) at carbon Vulcan support on the glassy carbon (GC) electrode (i.e., the substrate) without PPy, the redox interactions due to methanol were absent, Figure 5. In this context, an approach was carried out to measure the catalytic activity acat. (with methanol) or adsorption capacity (methanol free). This approach was based on the cyclic voltammetry experiments shown in Figure 5 and the amount of catalyst deposited at the working electrode WE [21], Equation (3).

(3) $$a_{cat.}=\frac{1}{z\cdot F\cdot m\cdot v}\cdot \int_{E_{0}}^{E}i\cdot dE$$

where z is the number of electrons transferred at solutions free of methanol (1e^−^, to accomplish OH- adsorption), and 6e^−^ during oxidation of methanol to CO_2_, F is the Faraday constant, m is the mass of catalysts deposited on the glassy carbon (GC) substrate, v is the scan rate (50 mV/s), i is the current intensity and E is the potential. The results of the adsorption capacity for the materials tested previously in CV without methanol and with z = 1 are depicted in Table 2. Similar trends were observed at solution containing 0.5 M of methanol (with z = 6), being Ni 8/PPy/C the most active sample for the reaction in study.

The findings in these results indicate a direct relation between adsorption activity and the weight % of Ni which constitutes the active phase (8% and 10%). The presence of PPy in the analyzed materials is a relevant factor in the relationship from an electrical conductivity perspective. This outcome is consistent with the chemical structure and stability of the polymer, which prevents chemical bonding between alkaline ions and the nitrogen N-sites which are present in the PPy molecular chains. Such electrical behavior was also verified using EIS technique.

**Figure 6:**
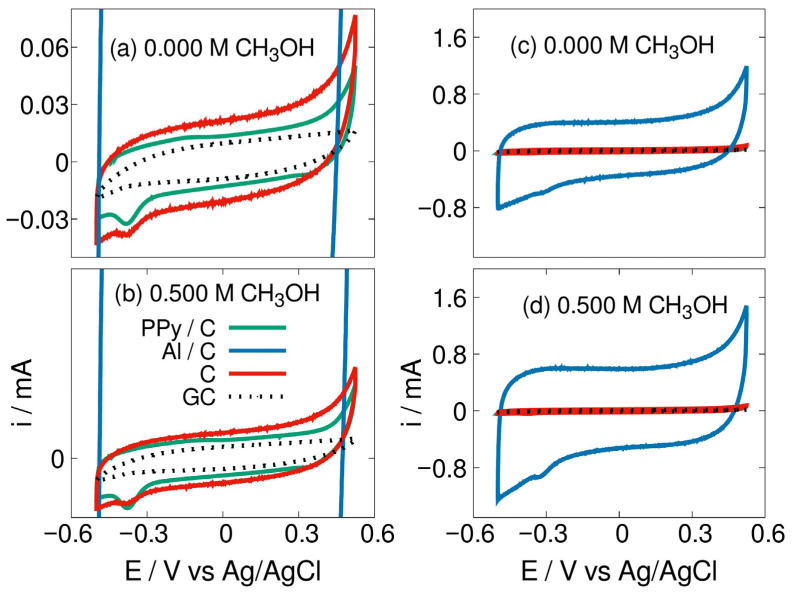
No-active-phase materials CV profiles, (**a**,**c**), without methanol, in 1.0 M KOH; (**b**,**d**) in 1.0 M KOH and 0.5 M methanol. Scan rate of 50 mV/s. Plots (**a**,**c**) are the same, but with different Y axis range; also (**b**,**d**) are the same.

Notable results are presented in Figure 6, where capacitive current intensity of Al/C material is highly different from the other shown. Lewis Al acidity provides Al/C high OH- adsorption capacity. Different capacitive phenomena are associated with PPy/C, which Lewis Al acidity is diminished, presumably after PPy anchorage: Lewis PPy basicity was thought to interact with Al atoms during synthesis, and is observed as a very lower OH- adsorption capacity.

Table 3 ECSA results show that surface active phase availability has been diminished due to the presence of PPy/Al(OH)_3_ combination. Particularly, this can be attributed to the remaining Lewis acidic properties of Al, which consequence is to possibly capture the OH- groups of nearby Ni(OH)_2_, limiting chemically and physically this active phase compound. Also due to the mentioned Lewis acidity, an excessive captivity of adsorbed OH- during activation could mean significant ECSA decrease compared to materials without PPy. Nevertheless, ECSA is not a property that definitively determines catalytic activity, but Rch. t. observed through EIS, as follows:

### 3.6. Electrochemical Impedance Spectroscopy (EIS)

The catalytic support materials at 8% and 10% of Ni active phase were analyzed by EIS to quantify the impedance changes during the methanol oxidation reaction. Following the systematic development of the analysis, the materials were tested by EIS in alkaline environment KOH without methanol, and then a series of assessments containing the methanol were carried out.

**Figure 7:**
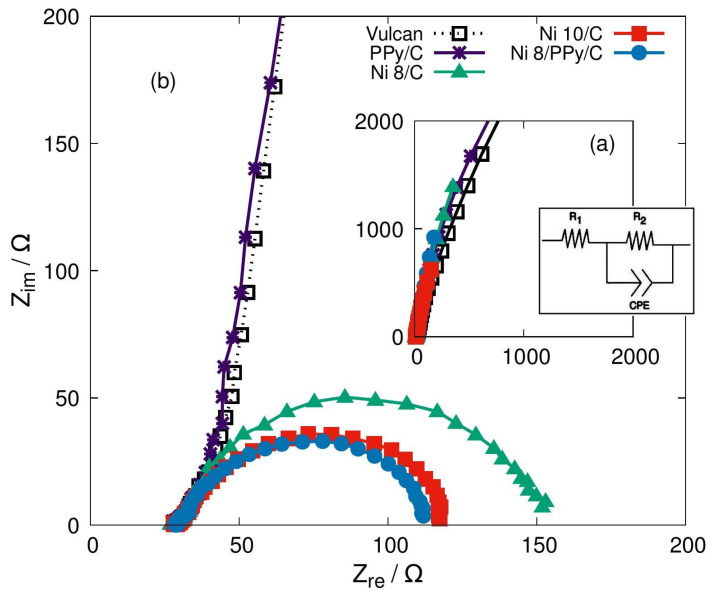
Nyquist profiles for each analyzed material in different systems. For (**a**) a 1.0 M KOH solution was used and for (**b**) a 1.0 M KOH with 0.5 M methanol solution was performed. All tests were developed at 0.375 V, the charge transfer process starting potential. Elements in circuit: R1, solution resistance; R2, charge transfer resistance; and CPE, the constant phase element.

For each profile the circuit in Figure 7 was proposed to determine the charge transfer resistance, Rch. t. equals to R2, after a previous data fitting to that model. In Figure 7 Rch. t. values for each material are compared while methanol is oxidized, observing for these cases that the lowest value corresponds to Ni 8/PPy/C catalyst that contains PPy. In Table 4 the numerical values corresponding to this comparison are presented, including also solution resistance Rsol. R1 in Figure 7 and CPE parameters.

The result can be explained considering that electric conduction enhancement is caused by interaction between carbon and PPy, the conducting materials of the proposed formulations. In fact, without PPy, Rch. t. lowering is observed directly dependent of active phase percentage; on the contrary, using PPy—that involves also Al(OH)_3_ usage—the same property does not show such a similar behavior. The Al(OH)_3_ altering the Ni(OH)_2_ as it has been mentioned and the exacerbated OH- adsorption during activation can explain that active phase percentage does not determine Rch. t. values. Nevertheless, the lowest resistance obtained was that using a high active phase quantity—not the highest, that is 10.0%—and adding some quantity of PPy with which involved Al(OH)_3_ could not interfere with the interest properties but enhance these with its adsorption capacity. After that, it is remarkable the influence of PPy on the electric conduction properties of the catalyst.

### 3.7. Chronoamperometry

**Figure 8:**
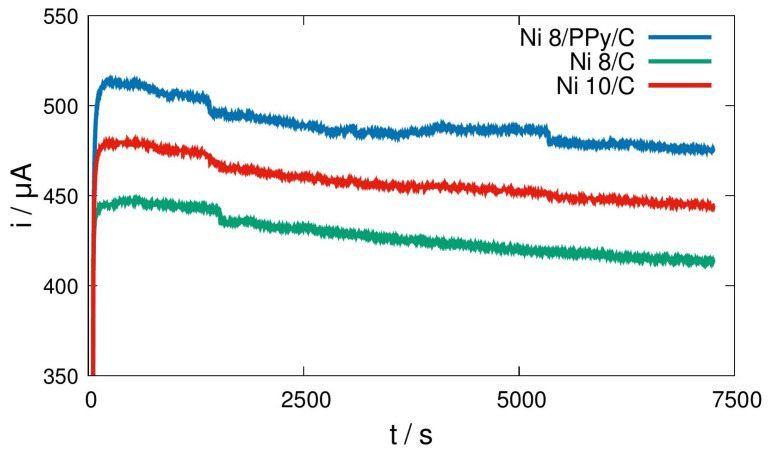
Chronoamperometry profiles observed at 0.375 V and 0.5 M methanol solution for Ni 8/PPy/C, Ni 8/C and Ni 10/C, with PPy as electric conduction enhancement additive.

As illustrated in Figure 8, the data demonstrate a variation in the behavior of 8.0% Ni-phase catalysts during the CA test. In particular, the catalysts under consideration are those containing PPy in the carbon support matrix, as well as those comprising Ni-metallic phase supported on carbon Vulcan. As anticipated, the current profile for the catalyst containing PPy is higher than for the Ni/C material free of the polymer part. This phenomenon can be attributed to the enhanced conducting properties exhibited by PPy, which consistently align with the results obtained from CV and EIS analyses.

Furthermore, an average current analysis has been conducted, incorporating current intensity and time data for both catalysts. This analysis yielded the reacting charge over time. The data obtained were then adjusted to a linear regression analysis, resulting in the determination of the first-order coefficients, see Table 5.

Also in Table 5, the drop in current intensity through time is presented. This drop is associated with active sites-redox stability at the applied potential. Therefore, elevated values for this decay are indicative of chemical catalyst instability. It has been demonstrated that materials containing PPy exhibit the lowest value of current decay, additionally, they are the most stable and offer high catalytic activity for methanol oxidation.

Another indicator of catalyst behavior is the TOF (turnover frequency), as shown in Equation (4). In this equation, i denote the average current intensity during reaction, z is the number of transferred electrons, F is the Faraday constant, and n is the molar mass of active sites (i.e., the proportional part of the active phase in the catalyst’s molar mass quantity) [22]. From this analysis the sample modified with PPy presented the highest TOF value, indicating a better probe-molecule consumption rate and, therefore, major catalyst stability.

(4) $$TOF=\frac{i}{z\cdot F\cdot n}$$

Following data so far given, benchmarking can be made as is shown in Table 6. Supporting electrolyte and electrolyte, the same as a consistent use of Ni as active phase remain similar to the present work. Onset potentials are also similar, almost the lowest for the Ni 8/PPy/C catalyst that has been observed as the most active in comparison to the also presented Ni 8/C and the Ni 10/C. Notorious is the stability value observed after Ni 8/PPy/C, which obtained from TOF-1 is about 2.08 × 10^5^ s is much superior to the rest in the table. In any case, aspects such as chemical steps followed during MOR, that are presented in the next section, are also important to consider the reasons behind catalytic activity and low Rch. t., the same as the effect these have in either complete or incomplete oxidation.

### 3.8. DEMS and RAMAN In Situ

**Figure 9:**
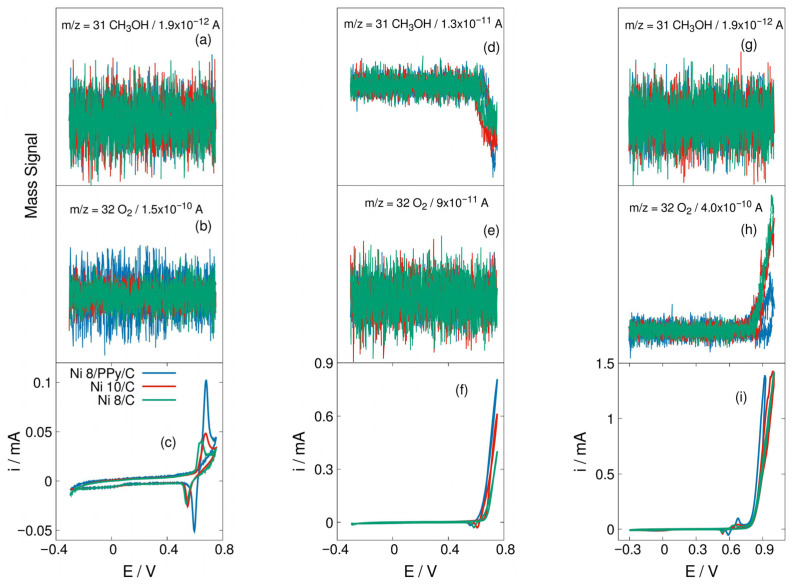
DEMS spectra using Ni 8/C, Ni 10/C and Ni 8/PPy/C catalysts, during 0.1 mV/s CV in a KOH 1.0 M solution; detecting *m*/*z* 31 (methanol)—(**a**,**d**,**g**), 32 (oxygen)—(**b**,**e**,**h**); from −0.30 to 0.75 V being (**c**) without methanol and (**f**) using a 0.5 M methanol solution; from −0.30 to 1.0 V being (**i**) without methanol.

The generation of mass spectra from the faradic-current profiles in Figure 9c,f,i was achieved through DEMS technique during cyclic voltammetry in presence and in absence of methanol, at scan rate of 1 mV/s. The objective of this study was to make a comparison between the potential employed for the afore mentioned CV profiles and a higher oxidation potential (1.0 V) with which oxygen was generated in absence of the probe molecule, Figure 9h. Interestingly, even though the faradic current is similar for the three materials, Figure 9i, DEMS shows that the OER (i.e., OH-/O_2_) is delayed to more positive potentials in the modified Ni/C matrix with PPy, see blue line in Figure 9i. The method demonstrates to assure the correspondence of former tests to methanol consumption, as evidenced by the decrease in the ionic current intensity at *m*/*z* = 31 [30,31], Figure 9d. This outcome is consistent with the anticipated result for all catalysts and was not detected in the absence of alcohol, c.f. Figure 9b,h, as expected. Furthermore, the results of the experiment demonstrated a direct correlation between the oxygen-kinetic reaction and the increment in the intensity of the ionic current *m*/*z* = 32 [32,33], Figure 9h at higher overpotentials in a methanol-free solution.

This finding is consistent with the established literature on the subject, which reports an increase in methanol consumption as a function of concentration and in the presence of metallic phase [30]. Indeed, the results obtained from the DEMS analysis indicate that all comparisons made for electrochemical tests are valid for methanol oxidation, with OER at more positive potentials, as observed in Figure 9d in a 0.5 M methanol solution.

Therefore, the highest methanol oxidation catalytic activity is associated with the material exhibiting the lower OER kinetic. The enhanced electronic interaction between the nickel-carbon matrix modified with PPy facilitates the modulation of the OER, which subsequently leads to a MOR conversion to CO_3_^=^ in the alkaline electrolyte (see below), as depicted by the reaction (7) [3,22].

(5) $${CH}_{3}OH+{2OH}^{-}\to HCOH+2H_{2}O+{2e}^{-}$$

(6) $${CH}_{3}OH+{4OH}^{-}\to HCOOH+3H_{2}O+{4e}^{-}$$

(7) $${CH}_{3}OH+{8OH}^{-}\to CO_{3}^{=}+6H_{2}O+{6e}^{-}$$

**Figure 10:**
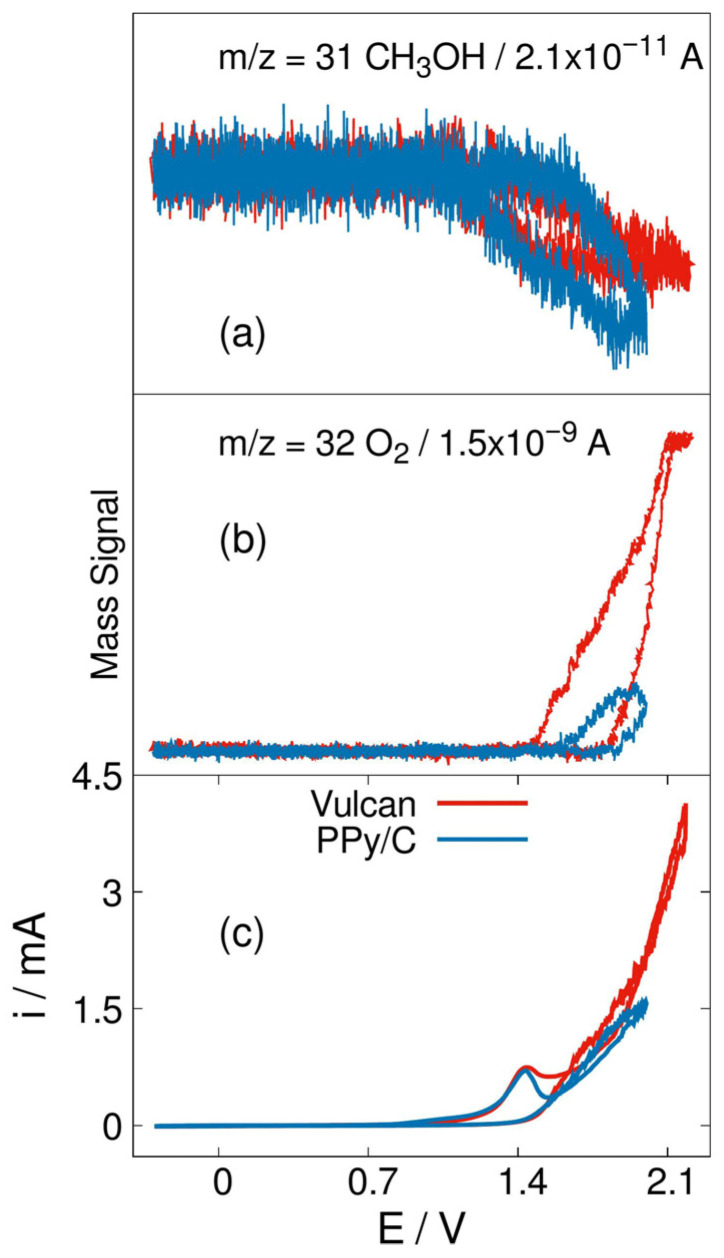
DEMS spectra from no-active-phase materials, which are carbon Vulcan and carbon Vulcan with PPy, using 0.1 mV/s CV in a KOH 1.0 M solution; detecting *m*/*z* 31 (methanol) (**a**) and 32 (oxygen) (**b**); from −0.30 to 2.10 V being using a 0.5 M methanol solution (**c**).

As OER kinetic is slower in Ni/C-PPy sample, Figure 10a, the interaction of hydroxyl ions with methanol at the electrode interface promotes its oxidation and then, a more marked consumption is observed when compared with the unmodified matrix.

**Figure 11:**
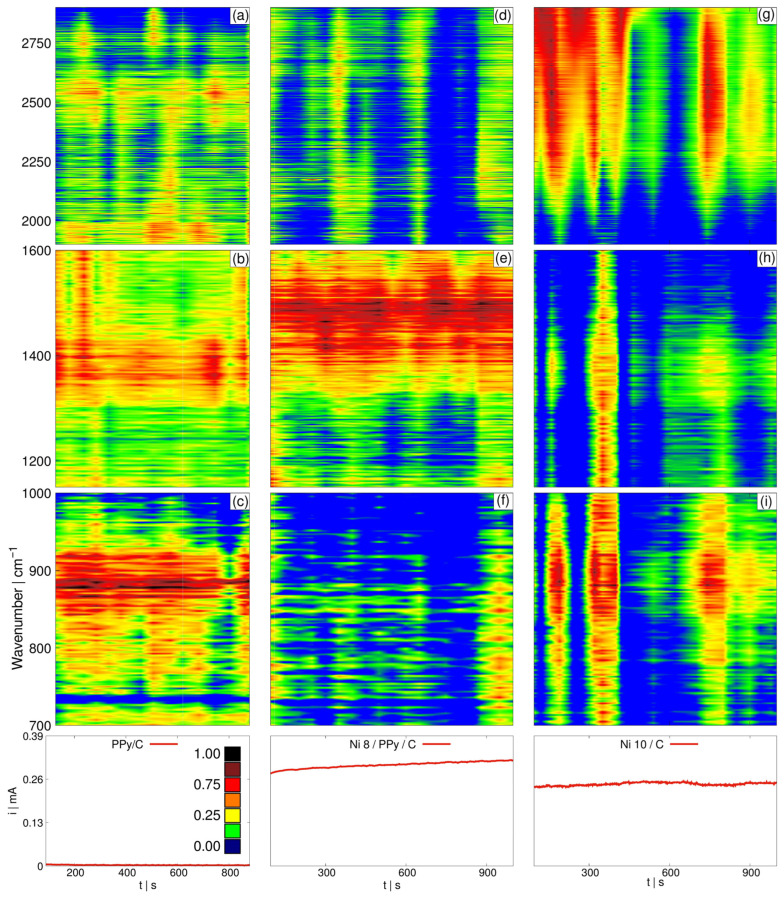
In situ Raman spectra as a set of heat maps for normalized intensities during 900 s CA and using a 0.5 M methanol solution for (**a**–**c**) anchored PPy on carbon, (**d**–**f**) Ni 8/PPy/C catalyst and (**g**–**i**) Ni 10/C catalyst. Red is for highest intensities, yellow and green for intermediate and blue for the lowest.

On the other hand, in Figure 11 Raman in situ spectra are presented. These profiles were obtained during chronoamperometry analysis at a constant potential of 0.375 V (the same used for EIS and CA tests) during 900 s, for unmodified and modified support matrix with PPy. A heat-map approach was used to distinguish different species generated-interacting at the electrode interface during anodic polarization. It is noteworthy that in the absence of active phase, see Figure 11a–c, the region ranging from ca. 800 to 950 cm^−1^ is marked in red, akin from 1315 to 1435 cm^−1^ region. The former is associated with the symmetric COC stretch vibrational mode [34], while the latter pertains to carboxylate salts [34] that may eventually transform into CO_3_ = ions [34]. On the other hand, for the metallic-phase modified with carbon-PPy, Figure 11d–f, the region from 1315 to 1435 and from 1405 to 1455 are flash lighted, indication of CH_3_ deformations in compounds linked with them and that are products of the MOR. Whereas species such as aldehydes, n-alkanes identified through symmetric CH_2_ stretch, and methoxy ions were detected at the unmodified Ni/C in the regions from 2680 to 2740, 2770 to 2830 and 2790 to 2850 cm^−1^, respectively [34], see Figure 11g–i, following the reaction processes in Equations (5) and (6). Consequently, the Raman in situ results demonstrate that the presence of PPy in Ni 8/PPy/C catalyst implies higher current intensities: according to Equation (7), more electrons are involved during complete MOR, than during incomplete oxidation processes. This, in turn, suggests higher catalytic activities and an increment in methanol consumption. On the other hand, Ni 10/C catalyst involves slower processes as generated intermediate species limit the overall rate during the redox process, as also demonstrated from EIS and DEMS analysis in this study.

Moreover, in the absence of an active phase, only carboxylate salts are observed, Figure 11c, showing low oxidation effect. Nevertheless, the intensity of COC vibrational mode, Figure 11e, indicates that oxidation is the main process occurring during polarization. For Figure 11f adsorption is very low, indicating that electro-oxidation reaction is faster due to better electric conductivity. This can be compared with Figure 11i in which some adsorbed species remain at the catalyst interface, thereby delaying methanol oxidation in comparison with the PPy-active phase containing sample.

## 4. Conclusions

The modified impregnation of chlorides and later hydroxylation synthesis served to obtain catalysts with nanometric crystallite sizes.

Using this method nickel (II) and aluminum hydroxides could be formed and attached to the carbon Vulcan support as they could be determined through XRD. No other chemical species, particularly oxides, were obtained as was demonstrated using XPS. SEM micrographs revealed that impregnation in tandem with coprecipitation as a synthesis method does not have influence on distribution of active catalytic species nor on morphology of supported materials. PPy electric conductivity advantages were observed according to the EIS low charge transfer resistance.

Synthesized materials were able to oxidize methanol under potential from 0.30 to 0.75 V vs. Ag/AgCl reference electrode. Methanol oxidation was reached in all cases where Ni(OH)_2_ was present. Cyclic voltammetry in the materials with Ni also confirmed the methanol oxidation. EIS Randles-circuit Nyquist profiles showed a low charge transfer resistance, Rch. t. It was found that the highest catalytic activity was obtained using Ni 8/PPy/C material.

DEMS and Raman in situ techniques showed that the influence of Rch. t. was determinant. DEMS allowed to conclude that methanol consumption was best oriented by the lowest Rch. t. catalyst, while oxygen generation, requiring less electrons, was best oriented by higher Rch. t. materials. Similarly, Raman spectroscopy via in situ for assessments in liquid environment revealed that a higher electric conduction for the Ni 8/PPy/C catalyst implied complete methanol oxidation. The products being carboxylate salts, eventually referred to CO_3_= in the alkaline solution. In this sense, selectivity can be directly related to electric conduction apported by the conducting polymer acting together with the also conducting support and the active phase.

## Figures and Tables

**Table 1:** Scherrer-equation-calculated crystallite sizes comparison in catalysts.

Catalyst	τ|nm
Ni 8/C	12.439
Ni 10/C	8.4089
Ni 8/PPy/C	4.1245

**Table 2:** Adsorption capacity and catalytic activity calculated from data (a) and (b) in Figure 5.

Catalyst	Ads. Cap.|nmol/μg	Cat. Act.|nmol/μg
Ni 8/C	0.1180	0.2835
Ni 10/C	0.1989	0.4309
Ni 8/PPy/C	0.1965	0.6075

**Table 3:** ECSA calculated for Ni 8/C, Ni 10/C and Ni 8/PPy/C catalysts.

Catalyst	ECSA|cm^2^ g^−1^
Ni 8/C	0.2835
Ni 10/C	0.4309
Ni 8/PPy/C	0.6075

**Table 4:** Fitting parameters calculated from data in Figure 6 and according to the proposed circuit. Notation: Rsol., solution resistance; Rch. t., charge transfer resistance; Q_0_ and *n*, the defining numbers of CPE.

Material	R_sol._|Ω	R_ch. t._|Ω	Q_0_|10^3^	*n*
Vulcan	29.250	18,380.0	1.260	0.92
PPy/C	29.250	17,100.0	1.030	0.89
Ni 8/C	31.239	116.0	4.081	0.92
Ni 10/C	29.250	108.0	6.200	0.76
Ni 8/PPy/C	30.904	84.8	4.904	0.82

**Table 5:** Average current and decay rate results, comparing all catalysts, with PPy and in the carbon-support matrix free of PPy.

Material	Average i|μA	Decay Rate|nA/s	TOF|μHz
Ni 8/PPy/C	487.7	−4.678	4.8016
Ni 8/C	426.6	−4.985	4.2001
Ni 10/C	457.0	−4.831	3.5996

**Table 6:** Benchmarking of materials, including the synthesized Ni 8/PPy/C, utilized for MOR in alkaline media [23].

Num.	Material	Electrolyte	CH_3_OH Conc.|M	E Onset|V vs. Ag/AgCl	Current Density	Stability|s
1	NCZMMO	1 M KOH	0.5	0.30	414 mA/mg	5.00 × 10^3^
2	NiCo_2_O_4_-rGO	1 M KOH	0.5	0.43	69.1 mA/mg	1.00 × 10^3^
3	CuO/Co (OH)_2_ (nanosheet)	1 M KOH	3	1.46	764 mA/mg	1.00 × 10^3^
4	NiO-MOF/rGO	1 M NaOH	3	1.63	275 mA/cm^2^	3.60 × 10^3^
5	GO/Co-MOF	1 M KOH	3	0.1	29.10 mA/cm^2^	3.60 × 10^3^
6	Ni@3DHPG	1 M KOH	0.75	0.63	147.108 mA/cm^2^	3.60 × 10^3^
7	NiO_AC@PPy	0.1 M KOH	0.5	0.5	551 mA/mg	1.00 × 10^4^
**8**	**Ni 8/PPy/C**	**1 M KOH**	**0.5**	**0.35**	**225.128 mA/mg**	**2.08 × 10^5^**
					**74.41 mA/cm^2^**	**(from TOF^−1^)**

Notation in table: NiO-MOF—nickel oxide metal–organic framework, Co-MOF—Cobalt metal–organic framework, Ni@3DHPG—Nickel and cobalt in situ grown 3D hierarchical porous graphene, rGO—reduced graphene oxide, NCZMMO—NiCoZn ternary mixed metal oxides. References for each material: 1 [24], 2 [25], 3 [26], 4 [27], 5 [28], 6 [29], 7 [23].

## Data Availability

The original contributions presented in this study are included in the article. Further inquiries can be directed to the corresponding authors.

## Abbreviations

The following abbreviations are used in this manuscript:

CA	Chronoamperometry
CPE	Constant Phase Element
CV	Cyclic Voltammetry
DEMS	Differential Electrochemical Mass Spectrometry
EDS	Energy-dispersive X-Ray Spectroscopy
EIS	Electrochemical Impedance Spectroscopy
GC	Glassy Carbon
IPD	Iodopyrrole Dimer
MOR	Methanol Oxidation Reaction
OER	Oxygen Evolution Reaction
PPy	Polypyrrole
SEM	Scanning Electron Microscopy
TEM	Transmission Electron Microscopy
TOF	Turnover Frequency
WE	Working Electrode
XPS	X-Ray Photoelectron Spectroscopy
XRD	X-Ray Diffraction
